# 1-(2-Methyl­phen­yl)-4,4′-bipyridin-1-ium tetra­fluorido­borate

**DOI:** 10.1107/S2414314622002486

**Published:** 2022-03-10

**Authors:** Claire E. Welton, Vladimir N. Nesterov, Bradley W. Smucker

**Affiliations:** a Austin College, 900 N Grand, Sherman, TX 75090, USA; b University of North Texas, 1155 Union Circle, Denton, TX 76203-5070, USA; Benemérita Universidad Autónoma de Puebla, México

**Keywords:** crystal structure, bipyridinium, hydrogen bond

## Abstract

Crystals of the title compound, C_17_H_15_N_2_^+^·BF_4_^−^, were unexpectedly grown from crystallization attempts of [Pt(4,4′-bpy)_4_](BF_4_)_2_ [Smith *et al.* (2019 ▸). *Comments Inorg. Chem.* **39**, 188–215] using toluene and aceto­nitrile. The tetra­fluoro­borate anion and the central pyridinium ring of the cation are disordered, with atomic site occupancies close to ½. The tolyl group of the cation has a 75.31 (11)° twist relative to the unsubstituted pyridyl group. This rotation allows for a centrosymmetric dimer of cations with weak hydrogen bonding between the pyridyl nitro­gen atom and a methyl H atom on the neighbouring cation.

## Abstract 2

In the crystal structure of the title compound, the cations pack as dimers connected by weak hydrogen bonds between the pyridyl nitro­gen and a methyl hydrogen atom on the neighbouring cation.

## Graphical abstract

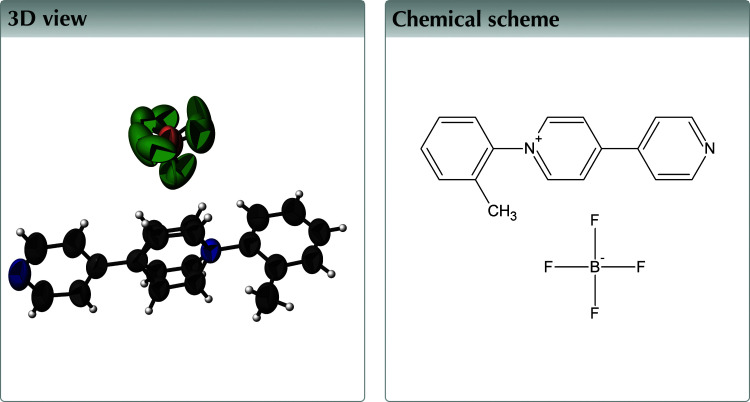

## Structure description

**Figure 1:**
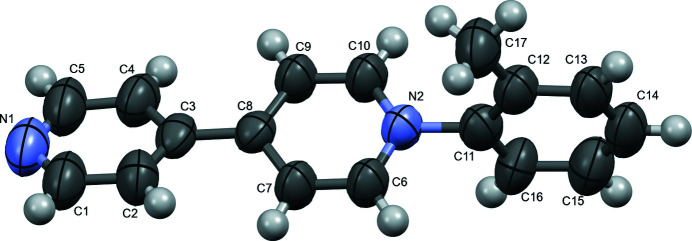
Ellipsoid (50% probability level) representation of the cation with disordered atoms omitted for clarity.

**Figure 2:**
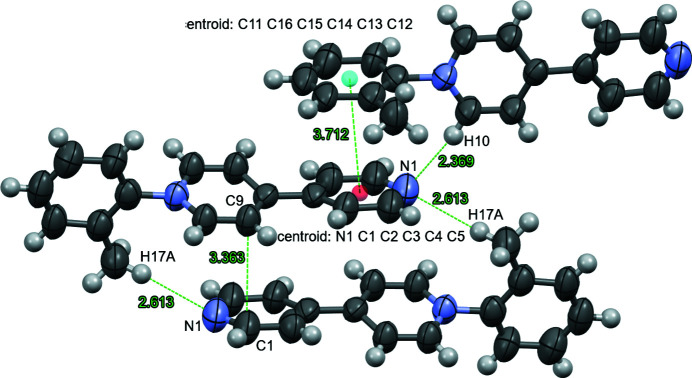
Ellipsoid (50% probability level) representation of the packing of the cations with the distances (Å) between the ring centroids of pyridyl-tolyl groups, N1⋯H10(− 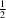 + *x*, 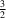 − *y*, − 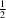 + *z*), H17*A*⋯N1(1 − *x*, 2 − *y*, 1 − *z*), and C9⋯C1(1 − *x*, 2 − *y*, 1 − *z*). Disordered atoms are omitted.

For each cation of the title structure, the ring of the tolyl group is twisted relative to the monosubstituted 4,4′-bipyridinium with a 75.31 (11)° rotation between planes (comprised of the tolyl ring (C11–C16) *versus* the unsubstituted pyrid­yl ring (N1/C1–C5) (Fig. 1 ▸) ; the central pyridinium ring (C6, C7, C9, C10) is disordered over two orientations with refined occupancies of 0.507 (6) and 0.493 (6). This twist is similar to the 78.12° between corresponding planes of a *N*-naphthyl monosubstituted 4,4′-bipyridinium cation (Lin & Zhao, 2015 ▸). The twisted conformation in the title compound allows for head-to-tail packing between two cations (Fig. 2 ▸). The mol­ecules in this dimer are slightly offset, which enables inter­molecular hydrogen bonding (H⋯N = 2.613 Å) between one cation’s methyl hydrogen, H17*A*, and N1 (1 − *x*, 2 − *y*, 1 − *z*) on the pyridyl group of the other cation (Table 1 ▸). The offset bipyridinium rings results in an inter­molecular C9⋯C1 (1 − *x*, 2 − *y*, 1 − *z*) distance of 3.363 (10) Å (Fig. 2 ▸). The twisted tolyl ring is face-to-face with a pyridyl group of another dimer (− 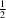 + *x*, 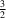 − *y*, − 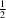 + *z*) at a distance (centroids of each ring) of 3.712 Å. The position of this adjacent dimer results in an N1⋯H10 (− 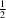 + *x*, 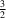 − *y*, − 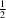 + *z*) distance of 2.369 Å between the pyridyl nitro­gen atom and the hydrogen atom on the other pyridinium ring (Fig. 2 ▸).

The C—N distance between the pyridinium and tolyl group is 1.487 (4) Å. This is longer than the C—N bond lengths observed in *N*-aryl structures of monosubstituted 4,4′-bipyridinium in: *N*-phenyl [1.460 (2) Å; Coe *et al.*, 1998 ▸], *N*-naphthyl [1.455 (2) Å; Lin & Zhao, 2015 ▸], or *N*-biphenyl [1.449 (5) Å; Schoder *et al.*, 2019 ▸]. The adjacent methyl group of the tolyl group is a likely factor for this longer C—N bond length, which is corroborated by the longer C—N bond distances of 1.463 (9) and 1.482 (9) Å resulting from an *ortho*-methyl group in the structure of the disubstituted *N*,*N*′-bis­(3-methyl-4-carboxyl­atophen­yl)-4,4′-bipyridinium bridging ligand (Wang *et al.*, 2020 ▸).

## Synthesis and crystallization

Colourless plate-shaped crystals of the title compound grew as a product from crystallization attempts using liquid diffusion of toluene into an aceto­nitrile solution of [Pt(4,4′-bpy)_4_](BF_4_)_2_ (Smith *et al.*, 2019 ▸).

## Refinement

Crystal data, data collection and structure refinement details are summarized in Table 2 ▸. In the crystal structure, both the BF_4_^−^ anion and four atoms of the central pyridinium ring (C6, C7, C9, C10) in the cation are disordered over two sets of sites, with a ratio of occupancies at *ca* 51 and 49%. These two occupancies of the pyridinium ring form a dihedral angle of about 30°. All our attempts to improve the quality of the refinement, such as disordering of the entire cation or only some of its rings, gave us similar results.

## Supplementary Material

Crystal structure: contains datablock(s) I. DOI: 10.1107/S2414314622002486/bh4067sup1.cif

Structure factors: contains datablock(s) I. DOI: 10.1107/S2414314622002486/bh4067Isup2.hkl

Supporting information file. DOI: 10.1107/S2414314622002486/bh4067Isup3.mol

Supporting information file. DOI: 10.1107/S2414314622002486/bh4067Isup4.cml

CCDC reference: 2156182

Additional supporting information:  crystallographic information; 3D view; checkCIF report

## Figures and Tables

**Table 1:** Hydrogen-bond geometry (Å, °)

*D*—H⋯*A*	*D*—H	H⋯*A*	*D*⋯*A*	*D*—H⋯*A*
C17—H17*A*⋯N1^i^	0.96	2.61	3.450 (6)	146

Symmetry code: (i) 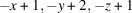 .

**Table 2:** Experimental details

Crystal data	
Chemical formula	C_17_H_15_N_2_^+^·BF_4_^−^
*M*_r_	334.12
Crystal system, space group	Monoclinic, *P*2_1_/*n*
Temperature (K)	200
*a*, *b*, *c* (Å)	11.4260 (5), 9.0735 (3), 15.5434 (5)
β (°)	102.118 (4)
*V* (Å^3^)	1575.54 (10)
*Z*	4
Radiation type	Cu *K*α
μ (mm^−1^)	1.00
Crystal size (mm)	0.10 × 0.09 × 0.01

Data collection	
Diffractometer	XtaLAB Synergy, Dualflex, HyPix
Absorption correction	Gaussian (*CrysAlis PRO*; Rigaku OD, 2019 ▸)
*T*_min_, *T*_max_	0.845, 1.000
No. of measured, independent and observed [*I* > 2σ(*I*)] reflections	16394, 2786, 2296
*R*_int_	0.023
(sin θ/λ)_max_ (Å^−1^)	0.595

Refinement	
*R*[*F* ^2^ > 2σ(*F* ^2^)], *wR*(*F* ^2^), *S*	0.108, 0.378, 1.63
No. of reflections	2786
No. of parameters	240
No. of restraints	30
H-atom treatment	H-atom parameters constrained
Δρ_max_, Δρ_min_ (e Å^−3^)	0.45, −0.24

Computer programs: *CrysAlis PRO* (Rigaku OD, 2019 ▸), *SHELXT2018/2* (Sheldrick, 2015*a* ▸), *SHELXL2018/3* (Sheldrick, 2015*b* ▸), *OLEX2* (Dolomanov *et al.*, 2009 ▸), and *Mercury* (Macrae *et al.*, 2020 ▸).

## full crystallographic data

### Crystal data

**Table:**

C_17_H_15_N_2_^+^·BF_4_^−^	*F*(000) = 688
*M**_r_* = 334.12	*D*_x_ = 1.409 Mg m^−^^3^
Monoclinic, *P*2_1_/*n*	Cu *K*α radiation, λ = 1.54184 Å
*a* = 11.4260 (5) Å	Cell parameters from 4873 reflections
*b* = 9.0735 (3) Å	θ = 4.4–74.8°
*c* = 15.5434 (5) Å	µ = 1.00 mm^−^^1^
β = 102.118 (4)°	*T* = 200 K
*V* = 1575.54 (10) Å^3^	Plate, colourless
*Z* = 4	0.10 × 0.09 × 0.01 mm

### Data collection

**Table:**

XtaLAB Synergy, Dualflex, HyPix diffractometer	2786 independent reflections
Radiation source: micro-focus sealed X-ray tube, PhotonJet (Cu) X-ray Source	2296 reflections with *I* > 2σ(*I*)
Mirror monochromator	*R*_int_ = 0.023
Detector resolution: 10.0000 pixels mm^-1^	θ_max_ = 66.6°, θ_min_ = 5.4°
ω scans	*h* = −13→13
Absorption correction: gaussian (*CrysAlis Pro*; Rigaku OD, 2019)	*k* = −8→10
*T*_min_ = 0.845, *T*_max_ = 1.000	*l* = −18→18
16394 measured reflections	

### Refinement

**Table:**

Refinement on *F*^2^	Primary atom site location: dual
Least-squares matrix: full	Secondary atom site location: difference Fourier map
*R*[*F*^2^ > 2σ(*F*^2^)] = 0.108	Hydrogen site location: inferred from neighbouring sites
*wR*(*F*^2^) = 0.378	H-atom parameters constrained
*S* = 1.63	*w* = 1/[σ^2^(*F*_o_^2^) + (0.2*P*)^2^] where *P* = (*F*_o_^2^ + 2*F*_c_^2^)/3
2786 reflections	(Δ/σ)_max_ < 0.001
240 parameters	Δρ_max_ = 0.45 e Å^−^^3^
30 restraints	Δρ_min_ = −0.24 e Å^−^^3^

### Fractional atomic coordinates and isotropic or equivalent isotropic displacement parameters (Å^2^)

**Table:**

	*x*	*y*	*z*	*U*_iso_*/*U*_eq_	Occ. (<1)
F1	0.3153 (14)	0.3155 (13)	0.6447 (8)	0.164 (4)	0.493 (6)
F2	0.4760 (14)	0.312 (2)	0.7610 (8)	0.169 (6)	0.493 (6)
F3	0.3583 (14)	0.524 (2)	0.7352 (10)	0.152 (5)	0.493 (6)
F4	0.4860 (11)	0.4406 (9)	0.6437 (8)	0.155 (4)	0.493 (6)
B1	0.415 (3)	0.402 (3)	0.6907 (18)	0.113 (3)	0.493 (6)
F1A	0.3290 (16)	0.530 (2)	0.7158 (11)	0.145 (5)	0.507 (6)
F2A	0.4771 (16)	0.372 (2)	0.7621 (12)	0.202 (8)	0.507 (6)
F3A	0.4091 (17)	0.418 (2)	0.6267 (7)	0.214 (9)	0.507 (6)
F4A	0.2994 (13)	0.3047 (13)	0.7003 (10)	0.193 (5)	0.507 (6)
B1A	0.380 (2)	0.409 (3)	0.7088 (17)	0.113 (3)	0.507 (6)
N1	0.2687 (4)	0.9335 (5)	0.3262 (2)	0.1134 (9)	
N2	0.4946 (2)	0.8531 (3)	0.78218 (17)	0.0764 (8)	
C1	0.2431 (4)	1.0258 (6)	0.3851 (3)	0.1134 (9)	
H1	0.190962	1.102669	0.364388	0.136*	
C2	0.2866 (4)	1.0187 (5)	0.4745 (3)	0.0982 (12)	
H2	0.264380	1.088352	0.511920	0.118*	
C3	0.3641 (3)	0.9056 (4)	0.5073 (2)	0.0768 (9)	
C4	0.3920 (4)	0.8102 (5)	0.4469 (2)	0.0979 (12)	
H4	0.443970	0.732164	0.465446	0.118*	
C5	0.3441 (4)	0.8285 (6)	0.3586 (3)	0.1134 (9)	
H5	0.366517	0.762100	0.319478	0.136*	
C6	0.3935 (8)	0.9217 (10)	0.7527 (5)	0.0836 (12)	0.493 (6)
H6	0.349547	0.957945	0.792095	0.100*	0.493 (6)
C7	0.3526 (8)	0.9402 (9)	0.6654 (5)	0.0836 (12)	0.493 (6)
H7	0.281258	0.991286	0.646357	0.100*	0.493 (6)
C8	0.4113 (3)	0.8872 (3)	0.60354 (19)	0.0694 (8)	
C9	0.5101 (8)	0.7906 (12)	0.6389 (7)	0.0836 (12)	0.493 (6)
H9	0.547025	0.737002	0.601039	0.100*	0.493 (6)
C10	0.5484 (9)	0.7783 (13)	0.7254 (8)	0.0836 (12)	0.493 (6)
H10	0.613277	0.717412	0.747470	0.100*	0.493 (6)
C11	0.5354 (3)	0.8302 (4)	0.8786 (2)	0.0833 (10)	
C12	0.6227 (3)	0.9222 (4)	0.9257 (2)	0.0893 (11)	
C13	0.6571 (4)	0.8949 (5)	1.0169 (2)	0.0947 (11)	
H13	0.716373	0.952582	1.051149	0.114*	
C14	0.6053 (4)	0.7857 (5)	1.0553 (2)	0.1002 (13)	
H14	0.629212	0.770262	1.115608	0.120*	
C15	0.5208 (5)	0.7005 (5)	1.0085 (3)	0.1115 (14)	
H15	0.486885	0.625935	1.036254	0.134*	
C16	0.4823 (4)	0.7217 (5)	0.9174 (2)	0.1013 (12)	
H16	0.422234	0.663387	0.884552	0.122*	
C17	0.6795 (5)	1.0384 (6)	0.8840 (3)	0.1146 (14)	
H17A	0.699061	1.001150	0.830901	0.172*	
H17B	0.751317	1.070252	0.923432	0.172*	
H17C	0.625551	1.120184	0.870080	0.172*	
C6A	0.5156 (8)	0.7563 (13)	0.7254 (8)	0.0836 (12)	0.507 (6)
H6A	0.560901	0.673187	0.745238	0.100*	0.507 (6)
C7A	0.4714 (8)	0.7748 (12)	0.6354 (7)	0.0836 (12)	0.507 (6)
H7A	0.486638	0.701903	0.597120	0.100*	0.507 (6)
C9A	0.3999 (7)	1.0067 (10)	0.6624 (5)	0.0836 (12)	0.507 (6)
H9A	0.367388	1.096731	0.640958	0.100*	0.507 (6)
C10A	0.4385 (7)	0.9833 (9)	0.7510 (5)	0.0836 (12)	0.507 (6)
H10A	0.426844	1.055966	0.790597	0.100*	0.507 (6)

### Atomic displacement parameters (Å^2^)

**Table:**

	*U*^11^	*U*^22^	*U*^33^	*U*^12^	*U*^13^	*U*^23^
F1	0.204 (9)	0.136 (6)	0.124 (7)	−0.009 (5)	−0.031 (7)	0.010 (6)
F2	0.148 (7)	0.277 (17)	0.081 (5)	0.003 (8)	0.023 (4)	0.057 (7)
F3	0.141 (8)	0.197 (11)	0.134 (10)	−0.064 (7)	0.068 (8)	−0.078 (8)
F4	0.212 (9)	0.108 (4)	0.184 (9)	0.036 (5)	0.129 (8)	0.021 (4)
B1	0.133 (14)	0.138 (5)	0.064 (10)	−0.010 (7)	0.013 (5)	0.003 (5)
F1A	0.168 (10)	0.168 (9)	0.093 (5)	0.019 (8)	0.011 (5)	0.007 (6)
F2A	0.160 (9)	0.250 (16)	0.165 (11)	0.061 (10)	−0.038 (7)	−0.008 (9)
F3A	0.270 (15)	0.282 (17)	0.102 (5)	0.184 (15)	0.066 (8)	0.042 (7)
F4A	0.208 (9)	0.140 (6)	0.200 (12)	−0.022 (5)	−0.026 (11)	−0.006 (8)
B1A	0.133 (14)	0.138 (5)	0.064 (10)	−0.010 (7)	0.013 (5)	0.003 (5)
N1	0.1264 (19)	0.146 (2)	0.0661 (14)	−0.0230 (14)	0.0169 (12)	−0.0021 (12)
N2	0.0827 (15)	0.0889 (17)	0.0585 (15)	0.0092 (12)	0.0171 (11)	−0.0079 (11)
C1	0.1264 (19)	0.146 (2)	0.0661 (14)	−0.0230 (14)	0.0169 (12)	−0.0021 (12)
C2	0.114 (3)	0.110 (3)	0.067 (2)	−0.004 (2)	0.0120 (18)	0.0072 (18)
C3	0.0793 (17)	0.093 (2)	0.0607 (18)	−0.0261 (15)	0.0203 (13)	−0.0015 (14)
C4	0.112 (3)	0.119 (3)	0.065 (2)	−0.007 (2)	0.0234 (19)	−0.0141 (18)
C5	0.1264 (19)	0.146 (2)	0.0661 (14)	−0.0230 (14)	0.0169 (12)	−0.0021 (12)
C6	0.096 (3)	0.093 (2)	0.0613 (12)	0.0127 (19)	0.0175 (19)	−0.0097 (14)
C7	0.096 (3)	0.093 (2)	0.0613 (12)	0.0127 (19)	0.0175 (19)	−0.0097 (14)
C8	0.0727 (15)	0.0772 (17)	0.0618 (18)	−0.0103 (12)	0.0219 (12)	−0.0022 (12)
C9	0.096 (3)	0.093 (2)	0.0613 (12)	0.0127 (19)	0.0175 (19)	−0.0097 (14)
C10	0.096 (3)	0.093 (2)	0.0613 (12)	0.0127 (19)	0.0175 (19)	−0.0097 (14)
C11	0.0872 (19)	0.095 (2)	0.068 (2)	0.0171 (16)	0.0167 (15)	−0.0106 (15)
C12	0.099 (2)	0.102 (2)	0.069 (2)	0.0096 (18)	0.0222 (17)	−0.0071 (16)
C13	0.106 (2)	0.116 (3)	0.061 (2)	0.027 (2)	0.0174 (17)	−0.0113 (18)
C14	0.126 (3)	0.113 (3)	0.063 (2)	0.032 (2)	0.023 (2)	−0.0025 (19)
C15	0.140 (3)	0.121 (3)	0.078 (2)	0.009 (3)	0.031 (2)	0.008 (2)
C16	0.129 (3)	0.115 (3)	0.063 (2)	0.000 (2)	0.029 (2)	0.0048 (18)
C17	0.128 (3)	0.137 (4)	0.074 (2)	−0.015 (3)	0.011 (2)	−0.004 (2)
C6A	0.096 (3)	0.093 (2)	0.0613 (12)	0.0127 (19)	0.0175 (19)	−0.0097 (14)
C7A	0.096 (3)	0.093 (2)	0.0613 (12)	0.0127 (19)	0.0175 (19)	−0.0097 (14)
C9A	0.096 (3)	0.093 (2)	0.0613 (12)	0.0127 (19)	0.0175 (19)	−0.0097 (14)
C10A	0.096 (3)	0.093 (2)	0.0613 (12)	0.0127 (19)	0.0175 (19)	−0.0097 (14)

### Geometric parameters (Å, º)

**Table:**

F1—B1	1.44 (3)	C7—C8	1.369 (8)
F2—B1	1.42 (3)	C8—C9	1.444 (11)
F3—B1	1.52 (3)	C8—C7A	1.271 (11)
F4—B1	1.248 (19)	C8—C9A	1.442 (8)
F1A—B1A	1.26 (3)	C9—H9	0.9300
F2A—B1A	1.28 (4)	C9—C10	1.329 (12)
F3A—B1A	1.39 (2)	C10—H10	0.9300
F4A—B1A	1.31 (3)	C11—C12	1.387 (5)
N1—C1	1.319 (6)	C11—C16	1.362 (5)
N1—C5	1.312 (7)	C12—C13	1.411 (5)
N2—C6	1.307 (8)	C12—C17	1.460 (6)
N2—C10	1.359 (11)	C13—H13	0.9300
N2—C11	1.487 (4)	C13—C14	1.356 (6)
N2—C6A	1.303 (11)	C14—H14	0.9300
N2—C10A	1.382 (8)	C14—C15	1.328 (6)
C1—H1	0.9300	C15—H15	0.9300
C1—C2	1.375 (6)	C15—C16	1.405 (5)
C2—H2	0.9300	C16—H16	0.9300
C2—C3	1.381 (5)	C17—H17A	0.9600
C3—C4	1.363 (5)	C17—H17B	0.9600
C3—C8	1.489 (4)	C17—H17C	0.9600
C4—H4	0.9300	C6A—C7A	1.393 (12)
C4—C5	1.376 (6)	C6A—H6A	0.9300
C5—H5	0.9300	C7A—H7A	0.9300
C6—H6	0.9300	C9A—C10A	1.372 (9)
C6—C7	1.350 (9)	C9A—H9A	0.9300
C7—H7	0.9300	C10A—H10A	0.9300
			
F2—B1—F1	106.1 (19)	C7A—C8—C9A	118.0 (6)
F2—B1—F3	104.7 (18)	C9A—C8—C3	119.5 (4)
F4—B1—F2	110 (2)	C8—C9—H9	119.9
F4—B1—F1	114 (2)	C10—C9—C8	120.1 (7)
F4—B1—F3	117 (2)	C10—C9—H9	119.9
F1—B1—F3	104.4 (19)	N2—C10—H10	119.4
F1A—B1A—F2A	122 (3)	C9—C10—N2	121.1 (8)
F1A—B1A—F3A	103 (2)	C9—C10—H10	119.4
F1A—B1A—F4A	108 (2)	C12—C11—N2	119.2 (3)
F2A—B1A—F3A	105.7 (17)	C16—C11—N2	118.2 (3)
F2A—B1A—F4A	112 (2)	C16—C11—C12	122.6 (3)
F4A—B1A—F3A	105 (2)	C11—C12—C13	116.3 (4)
C5—N1—C1	114.6 (4)	C11—C12—C17	122.5 (3)
C6—N2—C10	119.8 (6)	C13—C12—C17	121.2 (4)
C6—N2—C11	119.1 (4)	C12—C13—H13	119.5
C10—N2—C11	119.7 (6)	C14—C13—C12	121.1 (4)
C6A—N2—C11	121.7 (6)	C14—C13—H13	119.5
C6A—N2—C10A	118.5 (6)	C13—C14—H14	119.4
C10A—N2—C11	119.8 (4)	C15—C14—C13	121.2 (4)
N1—C1—H1	117.0	C15—C14—H14	119.4
N1—C1—C2	125.9 (5)	C14—C15—H15	119.6
C2—C1—H1	117.0	C14—C15—C16	120.7 (4)
C1—C2—H2	120.8	C16—C15—H15	119.6
C1—C2—C3	118.4 (4)	C11—C16—C15	118.1 (4)
C3—C2—H2	120.8	C11—C16—H16	120.9
C2—C3—C8	121.5 (3)	C15—C16—H16	120.9
C4—C3—C2	116.2 (3)	C12—C17—H17A	109.5
C4—C3—C8	122.2 (3)	C12—C17—H17B	109.5
C3—C4—H4	119.7	C12—C17—H17C	109.5
C3—C4—C5	120.5 (4)	H17A—C17—H17B	109.5
C5—C4—H4	119.7	H17A—C17—H17C	109.5
N1—C5—C4	124.3 (4)	H17B—C17—H17C	109.5
N1—C5—H5	117.9	C8—C7A—H7A	118.7
C4—C5—H5	117.9	C8—C7A—C6A	122.7 (8)
N2—C6—H6	119.8	N2—C6A—C7A	121.3 (9)
N2—C6—C7	120.4 (6)	N2—C6A—H6A	119.4
C7—C6—H6	119.8	N2—C10A—H10A	119.6
C6—C7—H7	118.5	C8—C9A—H9A	121.0
C6—C7—C8	123.0 (6)	C6A—C7A—H7A	118.7
C8—C7—H7	118.5	C7A—C6A—H6A	119.4
C7—C8—C3	122.6 (4)	C9A—C10A—N2	120.8 (6)
C7—C8—C9	113.9 (5)	C9A—C10A—H10A	119.6
C9—C8—C3	122.6 (5)	C10A—C9A—C8	117.9 (6)
C7A—C8—C3	122.2 (5)	C10A—C9A—H9A	121.0
			
N1—C1—C2—C3	−0.1 (6)	C7—C8—C9—C10	−10.3 (10)
N2—C6—C7—C8	1.4 (12)	C8—C3—C4—C5	178.2 (3)
N2—C11—C12—C13	179.8 (3)	C8—C9—C10—N2	1.4 (11)
N2—C11—C12—C17	−2.1 (5)	C8—C7A—C6A—N2	1.7 (11)
N2—C11—C16—C15	−179.9 (3)	C10—N2—C6—C7	−11.3 (11)
N2—C10A—C9A—C8	4.7 (11)	C10—N2—C11—C12	92.2 (6)
C1—N1—C5—C4	1.6 (7)	C10—N2—C11—C16	−89.8 (6)
C1—C2—C3—C4	0.7 (5)	C11—N2—C6—C7	−178.1 (6)
C1—C2—C3—C8	−177.6 (3)	C11—N2—C10—C9	176.6 (6)
C2—C3—C4—C5	−0.1 (5)	C11—N2—C6A—C7A	176.5 (5)
C2—C3—C8—C7	24.5 (6)	C11—N2—C10A—C9A	−179.9 (6)
C2—C3—C8—C9	−166.9 (6)	C11—C12—C13—C14	−1.1 (5)
C2—C3—C8—C7A	171.8 (6)	C12—C11—C16—C15	−1.9 (6)
C2—C3—C8—C9A	−14.9 (6)	C12—C13—C14—C15	0.4 (6)
C3—C4—C5—N1	−1.1 (7)	C13—C14—C15—C16	−0.4 (6)
C3—C8—C9—C10	−179.9 (5)	C14—C15—C16—C11	1.1 (6)
C3—C8—C7A—C6A	−179.7 (5)	C16—C11—C12—C13	1.9 (5)
C3—C8—C9A—C10A	176.6 (5)	C16—C11—C12—C17	180.0 (4)
C4—C3—C8—C7	−153.7 (6)	C17—C12—C13—C14	−179.2 (4)
C4—C3—C8—C9	14.9 (6)	C7A—C8—C9A—C10A	−9.8 (10)
C4—C3—C8—C7A	−6.4 (7)	C6A—N2—C11—C12	113.3 (6)
C4—C3—C8—C9A	166.9 (5)	C6A—N2—C11—C16	−68.7 (6)
C5—N1—C1—C2	−1.0 (7)	C6A—N2—C10A—C9A	3.6 (10)
C6—N2—C10—C9	9.9 (11)	C10A—N2—C11—C12	−63.2 (6)
C6—N2—C11—C12	−101.0 (6)	C10A—N2—C11—C16	114.9 (5)
C6—N2—C11—C16	77.0 (6)	C10A—N2—C6A—C7A	−7.0 (9)
C6—C7—C8—C3	178.7 (6)	C9A—C8—C7A—C6A	6.9 (10)
C6—C7—C8—C9	9.2 (11)		

### Hydrogen-bond geometry (Å, º)

**Table:**

*D*—H···*A*	*D*—H	H···*A*	*D*···*A*	*D*—H···*A*
C17—H17*A*···N1^i^	0.96	2.61	3.450 (6)	146

Symmetry code: (i) −*x*+1, −*y*+2, −*z*+1.
